# Optimized Partial Freezing Protocol Enables 10-Day Storage of Rat Livers

**DOI:** 10.21203/rs.3.rs-4584242/v1

**Published:** 2024-07-03

**Authors:** Ozge Sila Ozgur, McLean S. Taggart, Mohammedreza Mojoudi, Casie Pendexter, Anil Kharga, Heidi Yeh, Mehmet Toner, Alban Longchamp, Shannon N. Tessier, Korkut Uygun

**Affiliations:** Massachusetts General Hospital, Harvard Medical School; Massachusetts General Hospital, Harvard Medical School; Massachusetts General Hospital, Harvard Medical School; Massachusetts General Hospital, Harvard Medical School; Massachusetts General Hospital, Harvard Medical School; Massachusetts General Hospital, Harvard Medical School; Massachusetts General Hospital, Harvard Medical School; Massachusetts General Hospital, Harvard Medical School; Massachusetts General Hospital, Harvard Medical School; Massachusetts General Hospital, Harvard Medical School

## Abstract

Preserving organs at subzero temperatures with halted metabolic activity holds the potential to prolong preservation and expand the donor organ pool for transplant. Our group recently introduced partial freezing, a novel approach in high-subzero storage at −15°C, enabling 5 days storage of rodent livers through precise control over ice nucleation and unfrozen fraction. However, increased vascular resistance and tissue edema suggested a need for improvements to extend viable preservation.

Here, we describe an optimized partial freezing protocol with key optimizations including increased concentration of propylene glycol to reduce ice recrystallization and maintained osmotic balance through an increase in bovine serum albumin, all while minimizing sheer stress during cryoprotectant unloading with an acclimation period. These approaches ensured the viability during preservation and recovery processes, promoting liver function and ensuring optimal preservation. This was evidenced by increased oxygen consumption, decreased vascular resistance and edema.

Ultimately, we show that using the optimized protocol, livers can be stored for 10 days with comparable vascular resistance and lactate levels to 5 days, outperforming the viability of time-matched cold stored livers as the current gold standard. This study represents a significant advancement in expanding organ availability through prolonged preservation and thereby revolutionizing transplant medicine.

## Introduction

With more than five times the number of patients on the wait list than will receive a donor organ in the USA, the field of transplantation is facing a serious donor shortage crisis [1, 2]. Despite decades of research, a major limitation is the current preservation times for whole organs. The standard method, static cold storage (SCS) at 4°C, imposes a preservation time limit of 8–10 hours [3, 4]. Longer preservation times could have a profound impact on organ allocation, handling, and transplantation in various crucial ways. Firstly, prolonging the preservation duration would shift surgeries from emergency to planned, resulting in reduced transplantation costs and improved matching based on HLA compatibility [3, 5, 6]. Secondly, the protocols for inducing immune tolerance show promise in eliminating rejection and wanning patients off immunosuppressive regimen with numerous side effects including infection and organ failure, ultimately enhancing recipients’ quality of life, and improving patient survival. Thirdly, some organs that are procured for transplantation are discarded due to the complexity of allocation, that could be in theory eliminated with extended preservation duration [3, 4].

There are two divergent strategies applied in designing preservation methods for donor organs: metabolic support and metabolic depression. Metabolic support involves providing essential nutrients and oxygen to maintain the viability of organs. Machine perfusion, a cutting-edge advancement in this field, has emerged to both extend preservation duration and expand the donor pool of extended-criteria organ (ECD) [7], [8]. This process involves perfusing organs with various cellular and acellular media in a closed-loop, oxygenated circuit. Machine perfusion demonstrated stellar success in the preservation and recovery of potential grafts, increasing viable preservation time from approximately 12-hours, to up to 7-days [9], [10], [11]. Despite this success, limitations with the continuous perfusion method of preservation limit its successes including high costs, challenging logistics, and labor-intensive equipment [12], [13]. To combat these limitations, the field of cryopreservation aims to prolong liver preservation by lowering storage temperatures, and depressing metabolism beyond static cold storage.

Metabolic depression relies on slowing the depletion of energy stores [14], [15] by lowering temperature. While SCS at 4°C limits preservation time to 8–10 hours, further depression in temperature has the potential to allow indefinite storage (vitrification) [3, 16, 17]. Several preservation strategies were developed to take advantage of temperature-induced metabolic depression for the extension of organ storage [18–20]. For example, high-subzero techniques such as supercooling and isochoric preservation, whereby ice nucleation is avoided through careful maintenance of mechanical stability and volume, can facilitate multi-day storage [19, 21, 22]. Alternately, vitrification can stop biological time altogether, reaching temperatures as low as − 196°C, enabling indefinite storage [20]. However, these approaches are limited, with supercooling requiring highly stable storage conditions to prevent ice nucleation, isochoric preservation requiring technologically advanced storage systems for maintenance of constant volume, and vitrification requiring high-powered radiofrequency coils for rapid nanowarming to enable the warming rates necessary to avoid ice nucleation [23, 24].

Partial freezing (PF) is an attractive alternative, taking advantage of subzero preservation with controlled ice formation. Using extracellular ice nucleation mediated through specialized storage solutions, damaging intracellular ice formation can be avoided, enabling deeper storage temperatures than supercooling, as low as − 15°C [25], [26]. PF is a bioinspired technique derived from hibernation in the Sylvatica wood frog (*Rana Sylvatica*), which can survive in a frozen state at − 6°C to − 16°C for weeks. The wood frog capitalizes on both ice nucleating agents (INA) and endogenous CPAs to orchestrate freezing and prevent injurious intracellular ice formation [27], [28], [29]. The INAs promote ice formation within the vasculature as close as possible to melting point and the studies in freeze-tolerant species showed that controlled freezing of extracellular water by INAs is critical for freezing survival. As extracellular water gradually freezes, it is accompanied by an increase in the osmolality of the non-frozen extracellular fluid. This results in cellular dehydration as water is pulled from the intracellular environment. Another important strategy that confers freeze-tolerance is the synthesis of high amounts of carbohydrates, such as glucose. Glucose in the blood and tissues provides colligative resistance to detrimental decreases in cell volume and together with INAs restricts the formation of intracellular ice [30], [25]. Based on these observations, we successfully stored rat livers for 5 days using a combination of pressure-controlled machine perfusion to load and unload CPA/INA. Specifically, livers were gradually loaded with 3-O-methyl-D-glucopyranose (3-OMG; a glucose analog and metabolic depressor), SnoMax (a potent INA), polyethylene glycol 35k (PEG; a membrane stabilizer), trehalose (an extracellular cryoprotective agent (CPA), and propylene glycol (PG; an intracellular CPA) [31], [25]. However, using the current protocol, the extension of storage duration was limited by inadequate suppression of ice formation that results in elevated vascular resistance and graft edema, surrogates of poor outcome after transplantation.

Here, the study aimed to improve our PF protocol to extend liver preservation up to 10 days. To do so, we adopted the following changes: i) elevating PEG concentration to improve membrane stability, ii) increased BSA concentration to reduce edema upon rewarming, iii) the introduction of a 20-minute acclimation period during the thaw phase to limit sheer stress [32]. Liver viability after 5-day storage investigated during a 2-hour normothermic machine perfusion. After which, the efficacy of the improved protocol was tested for 10-day storage and compared to time-matched static cold storage using a blood-based simulated transplantation.

## Results

### 10% PEG Shows Improvements in Liver Function During Recovery

**Figure 1:**
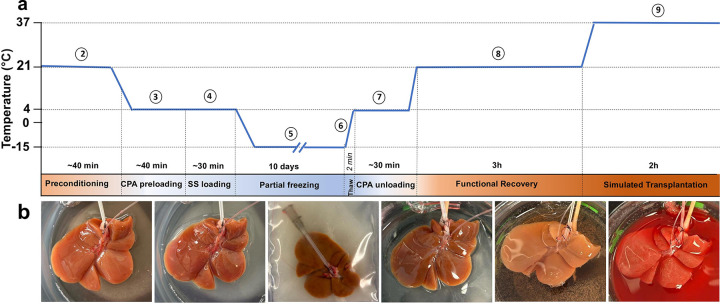
Experimental Design: **(a)** Schematic overview of rat liver partial freezing protocol showing 9 steps: (1) liver procurement, (2) preconditioning, (3) preloading CPAs, (4) loading of the final storage solution, (5) partial freezing, (6) thawing, (7) unloading CPAs, (8) functional recovery, (9) simulated transplantation or non-blood base NMP. The steps are outlined in Methods section with more details. The protocol was adapted from Tessier et al [25]. **(b)** Photos of liver during the consecutive steps of protocol. Left to right: SNMP preconditioning, CPA loading, partial freezing, CPA unloading, functional recovery during SNMP, simulated transplantation.

**Figure 2:**
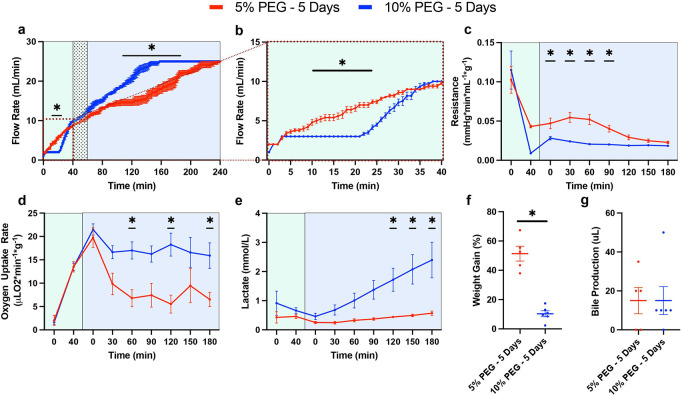
10% PEG Shows Improvements in Liver Function During SNMP Recovery: Comparison of liver functionality markers during the recovery phase following storage. Pressure driven flow led to a more rapid flow rate increase in the 10% PEG group (blue) compared to 5% PEG (red) during the subnormothermic recovery phase (SNMP, 21°C) **(a)**potentially driven by the inclusion of a 20-minute acclimation period during the hypothermic thaw phase (HMP, 4°C, green) **(b)**. Improved vascular health with 10% PEG is shown through decreased resistance early in the SNMP phase **(c)**. Elevated mitochondrial function is observed through increased oxygen uptake rate in the 10% PEG group throughout the SNMP phase **(d)**. Lactate showed faster elevation in the 10% PEG group **(e)**. Weight gain was markedly reduced at the end of SNMP in the 10% PEG group **(f).** No difference in bile production was observed between groups **(g).**

First, before viability testing during acellular NMP, perfusion metrics during the thaw (HMP) and recovery (SNMP) phases were collected to compare the original protocol (5% PEG) and the optimized protocol (10% PEG) (Presented in Fig. 1 and Supplementary Table S1). Perfusion pressure during HMP and SNMP were set at 3 mmHg and 5mmHg, with a maximum flow rate limit of 10 mL/min and 25 mL/min respectively. During and immediately after the added 20-minute acclimation phase, 5% PEG had an elevated flow rate from 10-min to 28 min when compared to 10% PEG, however, 10% PEG reached the same flow rate by 34 minutes (Fig. 2a–b). Importantly, compared to 5%PEG, 10%PEG led to a significant reduction in the vascular resistance (0.021 mmHg*min/L*g ± 0.005 vs 0.039 mmHg*min/L*g ± 0.016, p = 0.0054, Fig. 2c), an increase in oxygen uptake (17.5 uL O_2_/min*g ± 5.0 vs 9.3 uL O_2_/min*g ± 6.6, p = 0.015, Fig. 2d), but higher lactate production (1.38 mM ± 1.1 vs 0.38 mM ± 0.15, p = 0.0034, Fig. 2e). Moreover, end-perfusion weight gain was reduced with the 10% PEG (51.4% ± 11.4 vs 10.3% ± 5.0, p = 0.0042, Fig. 2f). Bile production was observed similar between the groups (15 uL ± 15 vs 15 uL ± 17.6, p = 0.7764, Fig. 2g).

10% PEG Partial Freezing Protocol Improves Liver Viability during acellular Normothermic Perfusion Following 5 Days of Storage

**Figure 3:**
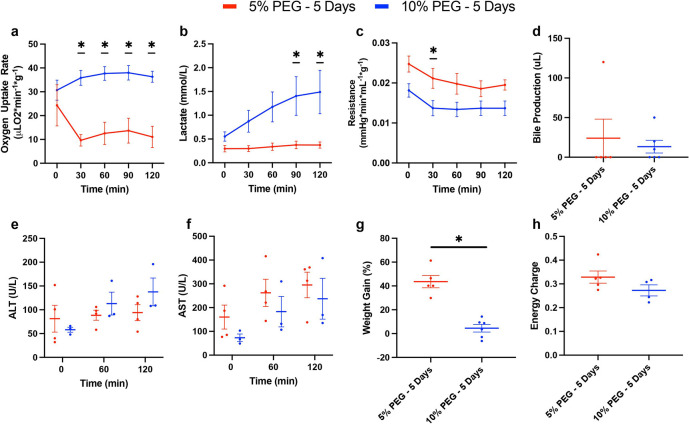
10% PEG Partial Freezing Protocol Improves Liver Performance under Normothermic Conditions Following 5 Days of Storage: To evaluate the performance of the partially frozen organs following storage in a more physiologically relevant manner, they were perfused with an acellular media at normothermic conditions (37C, NMP) for 2 hours. Oxygen uptake was greatly elevated in the 10% PEG group at 30, 60, 90, and 120 min **(a).** Lactate showed no change in 5% PEG, while a steady rise was observed in 10% PEG, resulting in significant elevation at 90 and 120 min **(b).** Resistance was similar throughout, showing the same dynamic trend, however, 5% PEG was significantly greater at 30 min **(c).** No difference in bile production was observed **(d).** Both ALT **(e)** and AST **(f)** showed the same trend in 10% PEG and 5% PEG, however, 10% PEG seemed to show a slight decrease. Weight gain was greatly reduced in 10% PEG, resulting in negligible edema **(g).** No difference in energy charge was shown **(h).**

**Figure 4:**
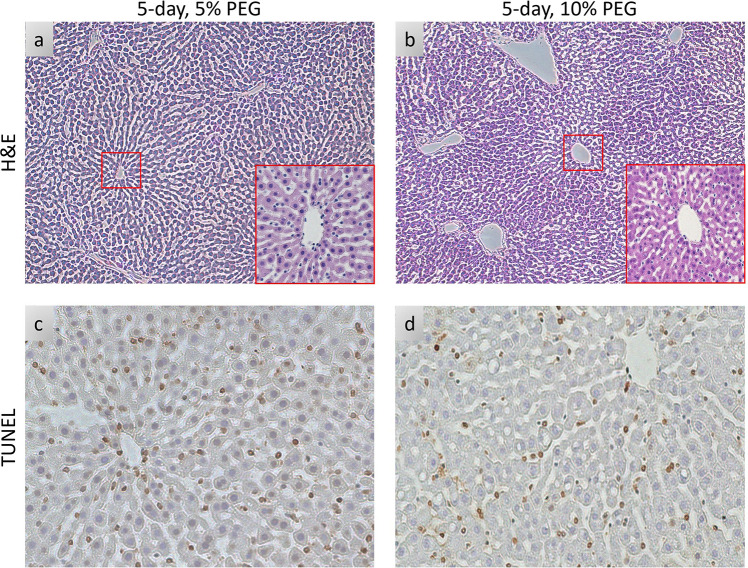
Histologic Comparison of 5% PEG and 10%PEG Partial Freezing: **(a, b)** Light microscopy images of parenchymal liver wedges at the end of NMP (10X, 20X), **(c, d)** Histological images with TUNEL staining at the end of NMP.

Since 10% PEG improved viability of livers during SNMP, we next investigated viability during a non-blood based, acellular NMP. 10% PEG improved oxygen uptake rate compared to 5% PEG (35.7 uL O_2_/min*g ± 7.7 vs 14.3 uL O_2_/min*g ± 12.4, p < 0.0001, Fig. 3a). Surprisingly, perfusate lactate steadily rose in the 10% PEG group, while 5% PEG remained low (1.10 mM ± 0.82 vs 0.34 mM ± 0.14, p = 0.0023, Fig. 3b). Resistance was lower in 10% PEG (0.015 mmHg*min/L*g ± 0.004 vs 0.021 mmHg*min/L*g ± 0.005, p = 0.0022, Fig. 3c). Bile production (Fig. 3d), damage markers ALT and AST (Fig. 3e/f), and energy charge (Fig. 3h) were similar between both groups (p = 0.7664, 0.5640, 0.3017, and 0.0676 respectively). Consistent with improved perfusion, weight gain was reduced in 10% PEG (3.6% ± 8.5 vs 43.6% ±11.6, p = 0.0008, Fig. 3g). This was confirmed on histological analysis; the lobular structure and LSECs were better preserved in 10% PEG group (Fig. 4a–b), whereas necrosis was similar between groups (Fig. 4c–d).

### Comparison of the Recovery Phase in Livers Partially Frozen for 5 and 10 Days

**Figure 5:**
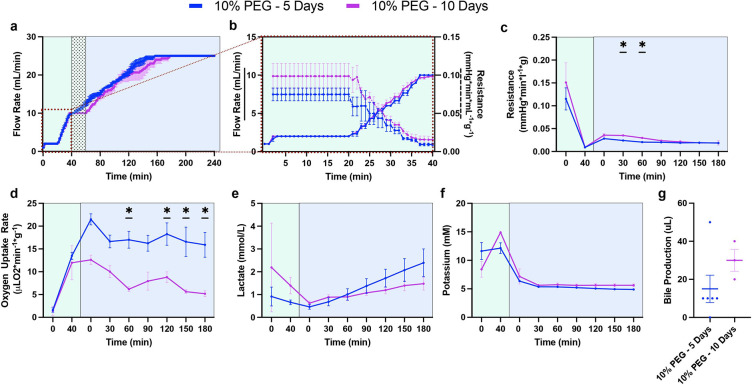
Comparison of the SNMP Recovery Phase in Livers Partially Frozen for 5 and 10 Days: Comparison of perfusion metrics between the recovery phase of rat livers stored using 10% PEG FOR 5- (PF5) and 10-days (PF10). Flow rate in PF5 and PF10 followed the same trend during SNMP **(a)** and, when looking at HMP, both resistance and flow rate followed the same trend **(b).** Resistance was elevated in PF10 during the beginning of SNMP at 30 min and 60 min, although it converged to meet PF5 **(c).** Oxygen uptake rate was decreased in PF10 throughout SNMP, showing significant reduction at 60 min, 120 min, 150 min, and 180 min **(d).** Lactate showed no difference, although PF5 seemed to show an increase in rate of elevation **(e).** No difference in potassium was observed **(f).** No difference in bile production was observed **(g).**

To determine the impact of 10-day storage period, liver perfusion was evaluated during thaw and SNMP recovery phase. During both thaw and SNMP, 5 mmHg pressure driven flow resulted in no difference in flow rate, with both PF5 and PF10 showing the same flow rate trend, with a slight, non-significant decrease in flow rate in PF10 from 130 min to 145 min (Fig. 5a). When isolating the HMP phase, no difference can be seen in either flow rate of resistance, showing overlapping trends in each, although a slight increase in resistance was seen during the 20-minute acclimation phase in PF10 (Fig. 5b). Resistance during SNMP was significantly higher after 10 days of storage compared to 5 days of storage at 30 min (0.035 mmHg*min/L*g ± 0.002 vs 0.024 mmHg*min/L*g ± 0.002, p = 0.0024) and 60 mins (0.030 mmHg*min/L*g ± 0.002 vs 0.021 mmHg*min/L*g ± 0.003, p = 0.0153), however, the resistances converged and no difference in average resistance was overserved (0.026 mmHg*min/L*g ± 0.008 for PF10 vs 0.021 mmHg*min/L*g ± 0.005 for PF5, p = 0.1429, Fig. 5c). A significant decrease in average oxygen uptake was observed in PF10 compared to PF5 (8.05 uL O_2_/min*g ± 3.01 vs 17.5 uL O_2_/min*g ± 5.3, p < 0.0001, Fig. 5d). Perfusate lactate rose steadily throughout recovery, but no difference was observed in both groups (0.011 ± 0.002 vs 0.005 ± 0.001, p = 0.0503, Fig. 5e). Similarly, potassium (5.13 mM ± 0.26 for PF5 vs 5.64 mM ± 0.36 for PF10, Fig. 5f) and bile production (15 uL ± 16.08 for PF5 vs 30 uL ± 10 for PF10, p = 0.6914, Fig. 5g) were similar.

#### Partial Freezing Improves Liver Function over Static Cold Storage Following in Simulated Transplantation.

**Figure 6:**
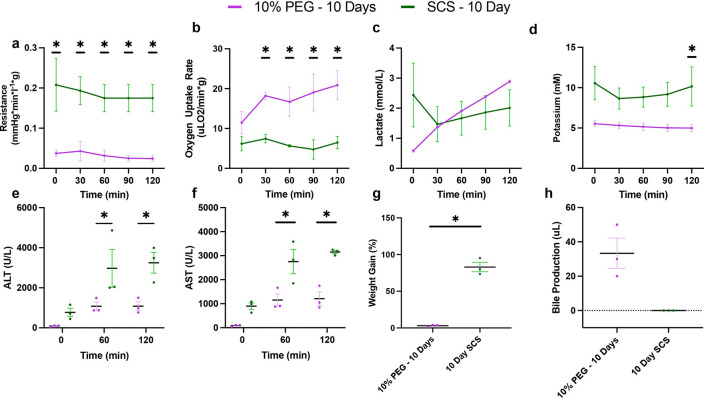
Partial Freezing Shows Vast Improvement in Simulated Transplantation Performance over Static Cold Storage Following 10 Days of Storage: Comparison of perfusion metrics during simulated transplantation between livers partially frozen (PF10) and static cold stored (SCS10) for 10 days. Resistance was significantly elevated throughout the entire simulated transplantation in SCS10 **(a).** Oxygen uptake rate was greater in PF10 at 30, 60, 90, and 120 min **(b).** No difference in lactate was observed, however, SCS10 showed slight elevation at 0 min **(c).** Potassium was elevated in SCS10 throughout, with a significant increase observed at 120 min **(d).** Both ALT **(e)** and AST **(f)** were significantly elevated at 60 min and 120 min. Weight gain was greatly elevated in SCS10, while PF10 showed next to no change in weight **(g).** Bile production was conserved in PF10, while SCS10 resulted in no bile production **(h).**

**Figure 7:**
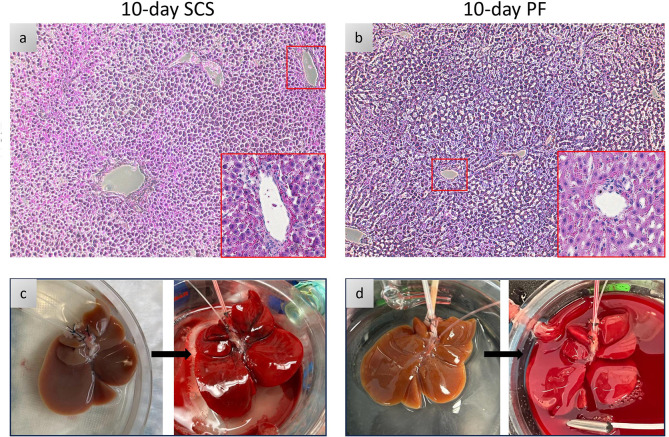
Microscopic and Macroscopic Evaluation of Livers Partially Frozen and Static Cold Stored for 10 Days: **(a, b)** Light microscopy images of parenchymal liver wedges at the end of simulated transplantation (x10, x20). **(c)** SCS liver, left: After 10-day cold storage, right: During simulated transplantation. **(d)** PF liver left: After 10-day freezing, right: During simulated transplantation.

Having demonstrated that 10% PEG improves liver viability during acellular perfusion, we next compared 10% PEG to the clinical gold standard (SCS) for 10 days during simulated transplant (Fig. 6). Vascular resistance was elevated following static cold storage at (0.032 mmHg*min/L*g ± 0.021 for PF10 vs 0.185 mmHg*min/L*g ± 0.063 for SCS10, p < 0.0001, Fig. 6a). Average oxygen uptake rate was higher in PF10 compared to SCS10 (17.3 uL O_2_/min*g ± 6.0 vs 6.1 uL O_2_/min*g ± 2.5, p < 0.0001, Fig. 6b). While lactate was lowest at T0 in PEG 10%, no difference in the average was seen over time (0.59 mM ± 0.09 for PF10 vs 2.44 mM ± 1.84 for SCS10, p = 0.0773, Fig. 6c). Outflow potassium was higher in SCS10 (9.48 mM ± 2.69 vs 5.20 mM ± 0.65, p < 0.0001, Fig. 6d), suggesting greater cellular damage. Consistently, liver transaminases (ALT and AST) were higher in SCS10 at 60 min (2973.67 U/L ± 869.28 vs 1078.67 U/L ± 356.23, p = 0.0401) and 120 min (3245.00 U/L ± 886.19 vs 1082.33 U/L ± 377.89, p = 0.0189, Fig. 6f). AST was also significantly elevated in SCS10 at 60 min (2753.33 U/L ± 869.28 vs 1150 U/L ± 444.48, p = 0.0031) and 120 min (3151.33 U/L ± 125.34 vs 1211.33 U/L ± 474.13, p = 0.0007, Fig. 6g). Importantly, edema post-transplant was 3% ± 2 in the PF10 compared to 83% ± 11, (p = 0.0064) in the SCS group (Fig. 6h). Bile production was sustained after PF10 but not SCS (33.33 uL ± 15.28 vs 0 uL ± 0, p = 0.0634, Fig. 6h). No difference was observed between PF10 and SCS10 in energy charge (0.23 ± 0.048 compared to 0.21 ± 0.022, p = 0.4386) **(Fig. S2l)**. Finally, the analysis of microscopic structure (Fig. 7a–b) showed greater disruption of hepatic lobular architecture in SCS10 compared to PF10. Furthermore, on the macroscopic level, SCS10 exhibited more edema and irregularly perfused regions across the liver, contrasting with the findings in PF10 (Fig. 7c–d).

## Discussion

Although the concept of preserving organs below freezing temperatures with halted metabolic activity is feasible, practical challenges hinder the anticipated level of viability. The storage at high subzero temperatures is limited by the destructive effects of ice formation, toxicity caused by high concentrations of CPA and challenges with rewarming [33, 34]. The major challenge in unoptimized partial freezing protocol was the excessive tissue edema that occurs on the course of the gradual switch of CPA unloading to recovery solution. Moreover, high initial vascular resistance during the recovery resulted in lower flow rates that precipitated decreased oxygen delivery to tissues thus eventually resulting in lower oxygen uptake rates. Inferred by these outcomes, we hypothesized that there is an underlying storage damage possibly caused by inadequate inhibition of ice crystallization. First, given that PEG is well-known for its ability to stabilize membranes and protects cells against freezing-induced damage caused by ice crystal growth[35, 36], we made an adjustment to the experimental protocol by elevating the concentration of PEG. We modified the concentrations by increasing PEG from 5–10% in final storage solution and from 1–2% in other solutions. Second, to minimize tissue edema by elevating vascular oncotic pressure, we increased BSA concentration in recovery solution from 1–7.5%. This aim further supported by incorporating a 20-minute acclimation period after the thaw phase. During this period, the liver is allowed to unload the CPAs by maintaining a stable flow rate of 2 mL/min and a portal pressure of 1–2 mmHg. We used a non-blood base solution for NMP following recovery period to rule out possible effects of whole blood on vascular structure while assessing the liver function.

Livers stored with this optimized protocol demonstrated a consistent portal pressure reduction while the gradual switch from the unloading solution to recovery solution. This allowed a quicker increase of the flow rates without any peak in the vascular resistance, ultimately resulted as significantly lower edema at the end of SNMP. As shown before with supercooled rat livers, the oxygen uptake is one of the indicators of post-transplant survival[22]. The oxygen uptake improved in 10% PEG group during SNMP and most significantly, in NMP phase. Conversely, lactate levels had an increasing pattern in 10% PEG group compared to steady levels of 5% PEG group, nevertheless, end NMP lactate levels of both groups were in the normal range (< 2.5 mmol/L)[37]. In means of hepatocellular injury, ALT levels were comparable between groups while AST levels were slightly lower in 10% PEG group. Tissue adenylate energy charge was comparable between groups, while ATP levels were higher in 10% PEG group. Histologic analysis with H&E staining showed greater cellular edema in 5% PEG group.

An increased concentration of PEG successfully mitigated the adverse effects of extracellular ice formation while 7.5% BSA maintained the oncotic pressure. This combination led to significant improvements in liver function, showing promising results. Leveraging these findings, we extended the storage duration to 10 days applying our enhanced protocol. To our knowledge, this is the first study testing 10-day high subzero storage, which is twice the duration of our group’s initial efforts. 10-day storage length has chosen to evaluate the suppression of metabolic activity when stored more than a week. While we assessed optimized 5-day (10% PEG) livers during acellular NMP phase, to evaluate the impact of longer storage duration we used simulated transplantation for 10-day livers.

Extending the storage duration from 5 days to 10 days led to high initial vascular resistance. However, by the end of SNMP, the vascular resistance in 10-day stored livers had decreased to the normal range and converged with 5-day (10% PEG) livers. The reduction in the oxygen uptake was significant in 10-day livers and lactate levels were comparable with 5-day (10% PEG) livers.

Our final investigation was to compare the viability of 10-day partially frozen livers (−15°C to −20°C) with 10-day static cold stored (4°C) livers during simulated transplantation. Partially frozen livers clearly outperformed SCS livers in all aspects. SCS livers gained excessive edema by the end (> 80%). Significantly reduced oxygen uptake and elevated potassium levels indicated cold ischemic damage in SCS group, when comparing with the high level of oxygen uptake and normal potassium levels in PF group. Regarding markers of hepatocellular injury, AST and ALT levels were notably lower in PF group. Even after freezing for 10 days, PF livers produced bile, this was not observed in SCS livers. Tissue adenylate energy charge was comparable between groups. Finally, SCS group showed greater destruction of hepatic structure on histology.

This study has its limitations. Future research endeavors involving large animal and discarded human liver studies that serves as a valuable alternative that approximates the conditions and challenges associated with transplant procedures. While these studies provide insightful data, transplantation procedures are crucial for understanding the practical implications of the partial freezing protocol.

In conclusion, we introduced an optimized partial freezing protocol to mitigate potential storage-related damage by precisely controlled ice formation. With these optimizations, we were able to demonstrate improved viability and functionality of livers up to 10 days storage.

## Methods

### Ethical statement

All the experimental protocols were approved by Institutional Animal Care and Use Committee (IACUC) of Massachusetts General Hospital (Boston, MA, USA; 2017N000227). All experiments were performed in accordance with relevant guidelines and regulations.

### Experimental Design

The experimental steps are shown in Figure 1 and adapted from Tessier et al. [25]; 1) liver procurement, 2) preconditioning during subnormothermic machine perfusion, 3) preloading CPAs during hypothermic machine perfusion, 4) loading of the final storage solution during HMP 5) freezing, 6) thawing, 7) unloading CPAs during HMP, 8) functional recovery during SNMP, 9) viability assessment during simulated transplantation or non-blood base NMP.

Our initial step was to replicate the same condition that has previously shown by our group, using 5% PEG in the storage solution. Next, we tested the effect of increased PEG concentration by 10% in storage solution, and 2% in preconditioning, loading and recovery solutions, and BSA concentration in recovery solution to 7.5%. Additionally, a 20-minutes acclimation period is introduced following the thawing phase. For this step, a total number of 10 rat livers were divided into 2 experimental groups (optimized/unoptimized) for 5-day storage duration (n = 5 per group). We excluded simulated transplantation to avoid the confounding effect of blood.

Once optimized, we evaluated the viability of PF livers stored for 10 days (n = 3) compared to time matched SCS (n = 3). Viability was evaluated with simulated transplantation using a blood base solution.

### Liver Procurement

Livers from healthy, adult female Lewis rats (10–12 weeks old, weighing 175–200 g) (Charles River Laboratories, Wilmington, MA, USA) were used for all experiments to ensure the consistency between groups. The animals were housed socially in a temperature and humidity-controlled room and provided unrestricted food and water.

The liver was procured as previously described [38]. The perfusion was started immediately after procurement, except for the static cold stored livers.

### Static Cold Storage

After procurement static cold stored livers were flushed with 30 ml of ice-cold Belzer UW^®^ Cold Storage Solution. Then livers were placed in a petri dish with 30 ml UW and stored at 4°C for 10 days.

### Machine Perfusion

Machine perfusion provides a continuous perfusion with pressure, flow, and temperature control through portal vein. The perfusion system setup and operation are previously described [38]. We used Radnoti (Cat# 130144) oxygenator.

### Partial Freezing Protocol

After procurement, livers were weighed and perfused with 250 ml of preconditioning solution (Supplementary Table S1) at 21°C, starting with the flow of 5 ml/min and gradually increased to 25 ml/min with the maximum pressure of 5 mmHg. Livers were perfused under this condition for 30 minutes, then preconditioning solution was gradually switched to preloading with 10% incremental increase in 25 ml volume. During the switch, temperature was set to 4°C and the flow rate was decreased to 7 ml/min to keep pressures below 3 mmHg and perfused for an additional 30 minutes. Next, preloading solution was gradually switched to storage solution with 10% incremental increase in 1 ml volume. The perfusion temperature was kept at 4°C and the flow rates were decreased to 1 ml/min for 5% PEG groups, and 0.5 ml/min for 10% PEG groups. The storage solution was loaded for 30 minutes, after which livers were placed in a bag with 25 ml of storage solution and immediately stored in a pre-cooled chiller at −15°C.

At the end of the storage period, the livers were removed from the bag and placed into a water bath (Cole Parmer, US) containing 25 ml of thawing solution at 37°C. The heater was turned off when the liver was placed, and the liver was gently swished until thawed, usually 2–3 minutes. Then the livers were connected to the perfusion system with the flow of 2 ml/min at 4°C for 20 minutes; within this period the flow rates in the unoptimized group were increased up to 10 ml/min with the maximum pressure of 3 mmHg, while the pressure was kept stable at 2 ml/min in the optimized group (acclimation period). After 20 minutes, temperature was gradually increased to 21°C with adjustments in flow rates to keep the pressure 4–5 mmHg. When the temperature reached to 21°C, the thawing solution gradually switched to recovery solution with 10% incremental increase in 25 ml volume.

After 100% recovery solution was reached, the liver was perfused with 250 ml of SNMP recovery solution for 3 hours with maximum pressure of 5 mmHg and the flow of 25 ml/min.

Following SNMP, 5-day (10% PEG) and 5-day (10% PEG) livers were removed and weighed, the system loaded with a fresh 250 ml recovery solution and temperature was set to 37°C. The livers were perfused for 2 hours with maximum pressure of 11 mmHg and flow of 30 ml/min (Fig. 1).

### Simulated Transplantation

After SNMP, 10-day (10% PEG) PF livers were removed from system and weighed. The perfusion system was emptied and loaded with 10% whole blood, %90 recovery solution with a total volume of 100 ml. Whole blood was obtained from one Lewis rat (~10 ml), stored at room temperature (21°C), and used within 4 hours after blood draw. For the control group, 10-day SCS livers were flushed with 30 ml of lactated ringer to rinse the UW out. Then livers were connected to the perfusion system. All livers perfused for 2 hours at 37°C with a flow rate of 30 ml/min or a maximum pressure of 11 mmHg.

### Viability Assessment

To measure edema, a precision pocket scale (MAXUS) was used, with weight measurements being taken immediately after surgery, prior to storage, after thawing, after recovery, and after NMP. Viability assessment was performed as described before [25].

### Histology

Wedge biopsies were taken at the end of NMP, and stored in 10% formalin which, after 24 hours, was moved to 70% ethanol. Samples were paraffin embedded, cut, and stained with Hematoxylin and eosin (H&E) and terminal deoxynucleotidyl transferase dUTP nick end labeling (TUNEL) as previously described [25].

### Liquid-Chromatography-Mass Spectrometry

To determine the concentration of various bioenergetic molecules, wedge biopsies of tissue was taken following NMP and was immediately flash frozen in liquid nitrogen. Samples were stored at −80C until analysis. Liquid-chromatography-mass spectrometry (LC-MS) was performed by the Mass Spectrometry Core Facility at Shriners Hospital (Boston MA, USA) as previously described [38]. Briefly, the tissue was homogenized in liquid nitrogen and analyzed with targeted multiple reaction monitoring.

### Statistical analysis

Statistical analysis and graphing were performed using GraphPad Prism 10 software version 10.0.1 (GraphPad Software, San Diego CA, USA). The two-sided significance was set to 0.05. Unpaired t-tests were performed on time-dependent data (results). Additional statistics (graphed) were performed using two-way analysis of variance (ANOVA) for time-dependent analysis, as well as post-hoc Tukey testing for significance. Statistics for time-independent data were determined using paired t-tests. All metrics are reported as means with standard deviation.

## Data Availability

The datasets generated during and/or analyzed during the current study are available from the corresponding author on reasonable request.
